# Supplementary material for: State-level macro-economic factors moderate the association of low income with brain structure and mental health in U.S. children
Source: Nat Commun. 2023 May 2;14:2085. doi: 10.1038/s41467-023-37778-1 (PMC10154403; doi:10.1038/s41467-023-37778-1)
Supplement: Supplementary file 1 — Supplementary Information [file 41467_2023_37778_MOESM1_ESM.pdf]

Supplemental Materials

Sensitivity Analyses

To determine if heterogeneity in the state-level associations between family income, hippocampal volume, and mental health was specifically related to cost of living and the generosity of anti-poverty programs, and not to other potentially correlated characteristics, we conducted sensitivity analyses controlling for 11 state-level social, economic, educational, and political characteristics, respectively, that may serve as alternative explanations. The rationale for selecting these specific characteristics is provided below. Because the number of these state-level characteristics is almost as high as the total number of states included in the ABCD sample, it was not possible to simultaneously control for all 11 characteristics. As such, we constructed a series of models that controlled for each of these characteristics individually in predicting hippocampal volume and internalizing problems. We examined whether our three-way interactions between family SES, cost of living, and generosity of anti-poverty policies remained significant after controlling for these state-level characteristics as a main effect (Table S2) and as a moderator of the association of SES with each of these outcomes (Table S3).

The measures of those characteristics were operationalized as follows, based on values at the time of data collection for the baseline ABCD sample.

Income inequality was operationalized by the state GINI index from the 2019 American Community Survey 5-year estimates.[1] The GINI index is a value between 0 and 1 indicating the distribution of economic resources in the population, where 0 indicates total economic equality and 1 indicates total economic inequality.[2]

Population density was calculated by dividing the estimated 2017 state population by the estimated land area of the state as reported by the U.S. Census Bureau.[1] Regions with greater

population density tend to have both higher costs of living and more generous antipoverty policies. However, the direct mechanisms through which population density may mitigate or augment the impacts of low SES are not currently known.

Unemployment is the average percentage of the labor force in the state that was unemployed in 2017 as reported by the U.S. Bureau of Labor Statistics.[4] Unemployment rates reflect economic conditions and would be expected to relate to family incomes and potentially to the generosity of anti-poverty policies.

Incarceration rate is the number of individuals incarcerated in the state out of every 100,000 in population as reported by the U.S. Bureau of Justice Statistics.[5] Because people who are incarcerated tend to come from lower SES backgrounds than those who are never incarcerated,[6] it is plausible that the impacts of low SES may be augmented in states that are more punitive and incarcerate a greater proportion of their population.

State Preschool Enrollment is the proportion of 4-year old children in each state enrolled in state-funded prekindergarten during the 2016-2017 school year as reported by the National Institute for Early Education Research.[7] It is plausible that providing preschool to a greater proportion of the population may mitigate the impacts of low SES on neurodevelopmental outcomes.

4th Grade Reading proficiency is the average reading score on the National Assessment of Educational Progress (NAEP) among students receiving free or reduced price lunch as reported by the National Center for Education Statistics.[8] It is plausible that these educational outcomes reflect the quality of the schools attended by low SES children in the state, potentially mitigating the impacts of low SES on neurodevelopmental outcomes.

Political preferences were estimated based on the results of the 2020 presidential election. These vales reflect the proportion of votes in each state that were cast for Joe Biden during the 2020 presidential election as reported by CNN and officially confirmed by the Federal Election Comission.[9,10] Regions that vote more Democratic tend to have both higher costs of living and more generous antipoverty policies. However, outside of a greater tendency to implement more generous antipoverty policies, the direct mechanisms through which political preferences may mitigate or augment the impacts of low SES on developmental outcomes are not currently known.

Women's political participation (Women's PP) is a composite score calculated by the Institute for Women's Policy Research[11] based on four indicators of women's political status: voter registration, voter turnout, representation in elected office, and women's institutional resources. Regions where women have higher political participation tend to have both higher costs of living and more generous antipoverty policies. However, the direct mechanisms through which women's political participation may mitigate or augment the impacts of low SES on developmental outcomes are not currently known.

Reproductive rights is a composite score calculated by the Institute for Women's Policy Research[11] based on nine indicators of women's reproductive rights: mandatory parental consent or notification laws for minors receiving abortions, waiting periods for abortions, restrictions on public funding for abortions, the percent of women living in counties with at least one abortion provider, pro-choice governors or legislatures, Medicaid expansion or state Medicaid family planning eligibility expansions, coverage of infertility treatments, same-sex marriage or second-parent adoption for individuals in a same-sex relationship, and mandatory sex education. Regions with greater reproductive rights tend to have both higher costs of living and more generous

antipoverty policies. Further, greater access to abortion, contraception, adoption, and sex education may directly impact family SES by increasing family size and needs. However, the potential role of reproductive rights in mitigating the impacts of SES on developmental outcomes is not currently known.

Tightness-looseness is a measure of the strength of punishment (tightness, e.g., the severity of punishment for violating laws) and the degree of permissiveness (looseness, e.g., access to alcohol) in a state.[12] Higher scores indicate greater tightness and lower looseness. Tightness-looseness has been found to be predict other state characteristics, such as rates of inequality, discrimination, social stability, and homelessness. However, the direct mechanisms through which tightness-looseness may augment or mitigate the impacts of low SES on developmental outcomes are not currently known.

Supplemental Tables and Figures

Table S1: Results of Models with only 2-way interactions between income and cost of living and income and the generosity of antipoverty programs

Hippocampal Volume

Cash Benefits				Medicaid Expansion			
	B	*SE*	*p*		*B*	*SE*	*p*
Income	60.5	7.42	<.001	Income	62.9	15.7	<.001
Cost of Living (COL)	-210	454	.650	COL	-.374	376	.999
Cash Benefit (Cash)	.0331	.227	.885	Medicaid Expansion (ME)	-24.0	34.6	.500
Income x COL	295	196	.133	Income x COL	-25.7	163	.875
Income x Cash	-.214	.108	.048	Income x ME	-1.36	21.6	.950

Internalizing Problems

Cash Benefits				Medicaid Expansion			
	B	*SE*	*p*		*B*	*SE*	*p*
Income	-.599	.150	<.001	Income	-.498	.292	.089
COL	3.57	9.83	.721	COL	3.67	8.67	.678
Cash	-.00417	.00556	.465	ME	-1.02	.907	.279
Income x COL	1.43	3.88	.712	Income x COL	3.48	3.00	.247
Income x Cash	.00088	.00217	.684	Income x ME	-.167	.400	.677

Externalizing Problems

Cash Benefits				Medicaid Expansion			
	B	*SE*	*p*		*B*	*SE*	*p*
Income	-1.52	.231	<.001	Income	-1.10	.456	.012
COL	-.495	7.59	.949	COL	-.785	6.38	.904
Cash	-.00371	.00428	.400	ME	-.721	.692	.314
Income x COL	6.14	5.86	.295	Income x COL	8.44	4.47	.059
Income x Cash	-.00047	.00330	.887	Income x ME	-.656	.592	.268

Note: Analyses were conducted using linear mixed-effects models with the nlme package in R using two-tailed tests. Age, sex, and the proportion of participants at each site that were White, Black, and Latinx were also included as covariates in all analyses.

Table S2: Results of moderation analyses examining the 3-way interaction between income, cost of living, and the minimum wage

	B	*SE*	*p*
**Hippocampal Volume**			
Income	82.1	9.18	<.001
Cost of Living (COL)	-120	400	.769
Minimum Wage (MW)	-2.44	9.02	.791
Income x COL	418	166	.012
Income x MW	-11.3	4.20	.007
COL x MW	53.4	150	.518
Income x COL x MW	-183	53.4	<.001
**Internalizing Problems**			
	B	*SE*	*p*
Income	-.916	.187	<.001
COL	13.5	9.21	.164
MW	-.538	.231	.035
Income x COL	.161	3.46	.963
Income x MW	.046	.087	.594
COL x MW	-1.45	3.22	.660
Income x COL x MW	2.70	1.08	.013
**Externalizing Problems**			
	B	*SE*	*p*
Income	-2.03	.325	<.001
COL	5.26	6.26	.415
MW	-.385	.229	.115
Income x COL	-5.11	5.09	.315
Income x MW	.280	.177	.113
COL x MW	-1.37	2.04	.512
Income x COL x MW	2.87	1.35	.033

Note: Analyses were conducted using linear mixed-effects models with the nlme package in R using two-tailed tests. Age, sex, and the proportion of participants at each site that were White, Black, and Latinx were also included as covariates in all analyses. *Minimum wage* is the states' minimum wage in 2017 as reported by the U.S. Department of Labor.[3] The federal minimum wage was used for states with no established minimum wage or with a minimum wage lower than the federal minimum wage.

Table S3. Summary of sensitivity analyses controlling for additional state-level characteristics

Control variable	Dependent Variable: Hippocampal Volume – Income x Cost of Living x Cash Benefits			Hippocampal Volume – Income x Cost of Living x Medicaid Expansion			Internalizing Problems – Income x Cost of Living x Cash Benefits			Internalizing Problems – Income x Cost of Living x Medicaid Expansion		
	B	*SE*	***p***	*B*	*SE*	***p***	*B*	*SE*	***p***	*B*	*SE*	*p*
Population Density	-3.64	1.16	**.002**	-974	290	**<.001**	.0614	.0232	**.008**	14.9	6.12	**.015**
Tightness	-3.62	1.16	**.002**	-976	298	**.001**	.0615	.0233	**.009**	15.1	6.22	**.015**
GINI coefficient	-3.61	1.16	**.002**	-977	297	**.001**	.0620	.0231	**.007**	15.0	6.16	**.015**
Political Preferences	-3.63	1.16	**.002**	-974	297	**.001**	.0606	.0229	**.008**	15.1	6.08	**.013**
Incarceration Rate	-3.62	1.16	**.002**	-978	297	**.001**	.0613	.0232	**.008**	14.9	6.17	**.016**
Unemployment Rate	-3.63	1.16	**.002**	-964	309	**.002**	.0614	.0231	**.008**	14.9	6.14	**.015**
State Preschool	-3.64	1.16	**.002**	-1000	312	**.001**	.0615	.0232	**.008**	14.9	6.16	**.015**
Women's PP	-3.59	1.16	**.002**	-983	292	**.003**	.0606	.0233	**.009**	15.1	6.16	**.015**
4th Grade Reading	-3.66	1.16	**.002**	-981	300	**.001**	.0613	.0231	**.008**	14.9	6.14	**.015**
Reproductive Rights	-3.72	1.16	**.001**	-993	297	**<.001**	.0615	.0231	**.008**	15.0	6.16	**.015**

Note: Analyses were conducted using linear mixed-effects models with the nlme package in R using two-tailed tests. Age, sex, and the proportion of participants at each site that were White, Black, and Latinx were also included as covariates in all analyses.

Table S4. Summary of sensitivity analyses controlling for state-level characteristics as moderators of the association of SES with hippocampal volume and internalizing problems

Control variable	Dependent Variable											
	Hippocampal Volume						Internalizing Problems					
	Income x Cost of Living x Cash Benefits			Income x Cost of Living x Medicaid Expansion			Income x Cost of Living x Cash Benefits			Income x Cost of Living x Medicaid Expansion		
	B	*SE*	***p***	*B*	*SE*	***p***	*B*	*SE*	***p***	*B*	*SE*	*p*
Population Density x Income	-3.49	1.18	**.003**	-931	293	**.002**	.0644	.0238	**.007**	15.7	6.44	**.015**
Tightness x Income	-4.00	1.22	**.001**	-1068	296	**<.001**	.0452	.0219	**.040**	13.5	6.07	**.027**
GINI coefficient x Income	-3.87	1.33	**.004**	-1012	306	**.001**	.0602	.0282	**.033**	13.9	6.64	**.036**
Political Preferences x Income	-3.79	1.30	**.004**	-974	305	**.001**	.0594	.0268	**.027**	15.2	6.38	**.017**
Incarceration Rate x Income	-2.91	1.51	.053	-945	321	**.003**	.0214	.0257	.407	11.0	5.94	.065
Unemployment Rate x Income	-3.91	1.28	**.002**	-952	317	**.003**	.0560	.0271	**.039**	14.2	6.23	**.023**
State Preschool x Income	-3.55	1.16	**.002**	-1025	315	**.001**	.0618	.0240	**.010**	15.1	6.42	**.019**
Women's PP x Income	-3.52	1.16	**.003**	-902	305	**.003**	.0626	.0224	**.005**	17.4	6.31	**.006**
4th Grade Reading x Income	-3.96	1.22	**.001**	-1042	531	**.049**	.0564	.0255	**.028**	18.5	10.4	.075
Reproductive Rights x Income	-3.82	1.17	**.001**	-937	335	**.005**	.0581	.0237	**.014**	18.0	6.26	**.004**

Note: Analyses were conducted using linear mixed-effects models with the nlme package in R using two-tailed tests. Age, sex, and the proportion of participants at each site that were White, Black, and Latinx were also included as covariates in all analyses.

Table S5. Results of sensitivity analyses with a dichotomous indicator for poverty

Hippocampal Volume							
Income dichotomized at the poverty line				Income dichotomized at <5x the poverty line			
	B	*SE*	*p*		*B*	*SE*	*p*
Poverty	-167	28.2	<.001	< 5x Poverty (Low Income)	-92.9	19.8	<.001
Cost of Living (COL)	-106	420	.803	Cost of Living (COL)	324	498	.525
Mean Cash Benefit (Cash)	.018	.211	.934	Mean Cash Benefit (Cash)	-.0308	.289	.917
Poverty x COL	-205	477	.668	Low Income x COL	-606	400	.129
Poverty x Cash	.0634	.279	.820	Low Income x Cash	.340	.235	.147
COL x Cash	1.99	2.76	.484	COL x Cash	-2.20	3.45	.533
Poverty x COL x Cash	6.39	3.26	.050	Low Income x COL x Cash	2.86	2.72	.294
	B	*SE*	*p*		*B*	*SE*	*p*
Poverty	-186	37.9	<.001	Low Income	-89.8	47.5	.059
COL	-223	610	.720	COL	338	919	.717
Medicaid Expansion (ME)	-6.73	40.3	.870	ME	17.6	63.5	.785
Poverty x COL	-1175	635	.065	Low Income x COL	-683	755	.365
Poverty x ME	48.9	46.3	.291	Low Income x ME	-2.64	51.6	.959
COL x ME	273	648	.680	COL x ME	-303	1009	.767
Poverty x COL x ME	1607	752	.033	Low Income x COL x ME	979	826	.236
**Internalizing Problems**							
Income dichotomized at the poverty line				Income dichotomized at <5x the poverty line			
	B	*SE*	*p*		*B*	*SE*	*p*
Poverty	2.07	.519	<.001	Low Income	1.08	.346	.002
COL	3.35	10.5	.755	COL	-1.99	8.51	.819
Cash	-.00461	.00601	.456	Cash	.00198	.00670	.771
Poverty x COL	3.33	8.96	.710	Low Income x COL	.133	5.74	.982
Poverty x Cash	-.00404	.00518	.485	Low Income x Cash	-.00498	.00466	.285
COL x Cash	.0420	.0819	.616	COL x Cash	.00840	.0548	.880
Poverty x COL x Cash	-.123	.0618	.047	Low Income x COL x Cash	-.00775	.0310	.802
	B	*SE*	*p*		*B*	*SE*	*p*
Poverty	2.82	1.00	.005	Low Income	2.31	.953	.015
COL	1.15	14.4	.938	COL	.519	13.1	.969
ME	-.948	1.43	.518	ME	-.420	1.27	.744
Poverty x COL	16.3	11.3	.148	Low Income x COL	9.14	10.4	.378
Poverty x ME	-1.04	1.13	.361	Low Income x ME	-1.37	1.01	.174
COL x ME	-.660	16.2	.968	COL x ME	2.18	14.0	.878
Poverty x COL x ME	-27.2	12.5	.030	Low Income x COL x ME	-14.9	11.0	.177

Note: Analyses were conducted using linear mixed-effects models with the nlme package in R using two-tailed tests. Age, sex, and the proportion of participants at each site that were White, Black, and Latinx were also included as covariates in all analyses.

Table S6: Results of moderation analyses including only the largest site in Florida and California

Hippocampal Volume

Cash Benefits	*B*	*SE*	*p*	Medicaid Expansion	*B*	*SE*	*p*
Income	79.0	9.95	<.001	Income	75.7	19.4	<.001
Cost of Living (COL)	-356	621	.579	COL	-741	864	.412
Cash Benefit (Cash)	.0852	.322	.797	Medicaid Expansion (ME)	16.7	56.9	.775
Income x COL	138	194	.478	Income x COL	475	292	.104
Income x Cash	-.0115	.114	.920	Income x ME	-4.91	21.6	.820
COL x Cash	3.39	2.91	.271	COL x ME	754	813	.376
Income x COL x Cash	-3.10	1.26	.014	Income x COL x ME	-524	274	.063

Internalizing Problems

Cash Benefits	*B*	*SE*	*p*	Medicaid Expansion	*B*	*SE*	*p*
Income	-.913	.201	.001	Income	-1.01	.434	.021
COL	5.13	13.4	.711	COL	31.0	23.0	.208
Cash	-.00482	.00818	.569	ME	-2.84	1.73	.132
Income x COL	1.54	3.90	.692	Income x COL	-7.60	6.46	.240
Income x Cash	-.00093	.00230	.685	Income x ME	.234	.477	.623
COL x Cash	.0213	.0943	.827	COL x ME	-28.1	24.2	.274
Income x COL x Cash	.0561	.0263	.033	Income x COL x ME	13.0	7.30	.075

Note: Age, sex, and the proportion of participants at each site that were White, Black, and Latinx were also included as covariates in all analyses.

Table S7. Distribution of family income across the States in the ABCD Sample

State	Mean Income-to-needs ratio	Below poverty*	1-2x poverty*	2-5x poverty*	>5x poverty*
CA	3.76	.20	.14	.32	.34
CO	4.42	.05	.10	.47	.39
CT	4.15	.15	.10	.38	.38
FL	2.55	.29	.22	.35	.14
MD	3.79	.22	.09	.33	.35
MI	3.80	.14	.13	.43	.31
MN	4.49	.03	.10	.46	.40
MO	3.09	.18	.17	.48	.17
NY	3.61	.21	.10	.34	.35
OK	2.72	.24	.22	.37	.17
OR	4.32	.06	.11	.44	.39
PA	1.80	.44	.26	.23	.08
SC	3.71	.16	.15	.36	.33
UT	3.12	.09	.22	.56	.13
VA	3.45	.16	.13	.48	.24
VT	4.70	.04	.08	.39	.49
WI	4.15	.09	.07	.50	.34

*Numbers represent the proportion of the sample in that state within that income bin.

Table S8. Participant Exclusion by State

State	Outcome	Participants with data	# excluded for poor data quality	# Missing data on income	% Excluded from Analyses
CA	Hippocampal Volume	1800	100	285	20.6
	Psychopathology	1933		304	15.7
CO	Hippocampal Volume	554	4	32	6.5
	Psychopathology	565		33	5.8
CT	Hippocampal Volume	620	18	81	15.5
	Psychopathology	634		83	13.1
FL	Hippocampal Volume	1075	7	146	14.2
	Psychopathology	1085		149	13.7
MD	Hippocampal Volume	591	18	91	17.6
	Psychopathology	605		96	15.9
MI	Hippocampal Volume	706	96	44	19.0
	Psychopathology	722		47	6.5
MN	Hippocampal Volume	595	7	29	6.1
	Psychopathology	607		30	4.9
MO	Hippocampal Volume	684	18	47	9.5
	Psychopathology	705		48	6.8
NY	Hippocampal Volume	339	8	44	15.0
	Psychopathology	339		44	13.0
OK	Hippocampal Volume	734	87	78	21.4
	Psychopathology	743		78	10.5
OR	Hippocampal Volume	582	4	41	7.7
	Psychopathology	585		42	7.4
PA	Hippocampal Volume	427	24	75	22.0
	Psychopathology	454		81	17.8
SC	Hippocampal Volume	375	7	52	15.2
	Psychopathology	376		5	13.3
UT	Hippocampal Volume	1000	6	30	3.5
	Psychopathology	1002		30	3.0
VA	Hippocampal Volume	538	27	61	15.2
	Psychopathology	552		62	11.2
VT	Hippocampal Volume	573	23	36	9.9
	Psychopathology	578		36	6.2
WI	Hippocampal Volume	340	28	20	14.1
	Psychopathology	385		23	6.0

Table S9. Correlations

	1	2	3	4	5	6	7	8	9
1. Sex (female)	-								
2. Age	-.021	-							
3. Log income-to-needs ratio	-.004	.038	-						
4. Cash assistance	-.003	-.007	.113	-					
5. Cost of living	-.003	-.008	.011	.779	-				
6. Medicaid expansion	.005	.028	.110	.687	.564	-			
7. Hippocampal volume	-.305	.065	.202	-.018	.009	-.020	-		
8. Internalizing problems	-.090	.008	-.065	-.044	-.022	-.065	.009	-	
9. Externalizing problems	-.080	-.029	-.160	-.056	-.022	-.062	-.049	.588	-

Table S10. Results for 3-way interactions when including only one participant per family

**Hippocampal Volume**							
	B	*SE*	*p*		*B*	*SE*	*p*
Income x Cost of living x Cash Benefits	-3.65	1.27	.004	Income x Cost of Living x Medicaid Expansion	-1074	311	<.001
**Internalizing Problems**							
	B	*SE*	*p*		*B*	*SE*	*p*
Income x Cost of Living x Cash Benefits	.0566	.0205	.006	Income x Cost of Living x Medicaid Expansion	13.4	5.12	.009

Note: Analyses were conducted using linear mixed-effects models with the nlme package in R using two-tailed tests. Age, sex, and the proportion of participants at each site that were White, Black, and Latinx were also included as covariates in all analyses.

Table S11. Results for 3-way interactions when controlling for race and ethnicity at the individual level

Hippocampal Volume							
	B	*SE*	*p*		*B*	*SE*	*p*
Income x Cost of living x Cash Benefits	-3.75	1.27	.003	Income x Cost of Living x Medicaid Expansion	-1235	312	<.001
**Internalizing Problems**							
	B	*SE*	*p*		*B*	*SE*	*p*
Income x Cost of Living x Cash Benefits	.0577	.0205	.005	Income x Cost of L x ME	13.5	5.17	.009

Note: Analyses were conducted using linear mixed-effects models with the nlme package in R using two-tailed tests. Age, sex, and the proportion of participants at each site that were White, Black, and Latinx were also included as covariates in all analyses.

Figure S1. Distribution of Cost of Living Relative to Mean Cash Benefits and Centering For Simple Slopes Analysis.

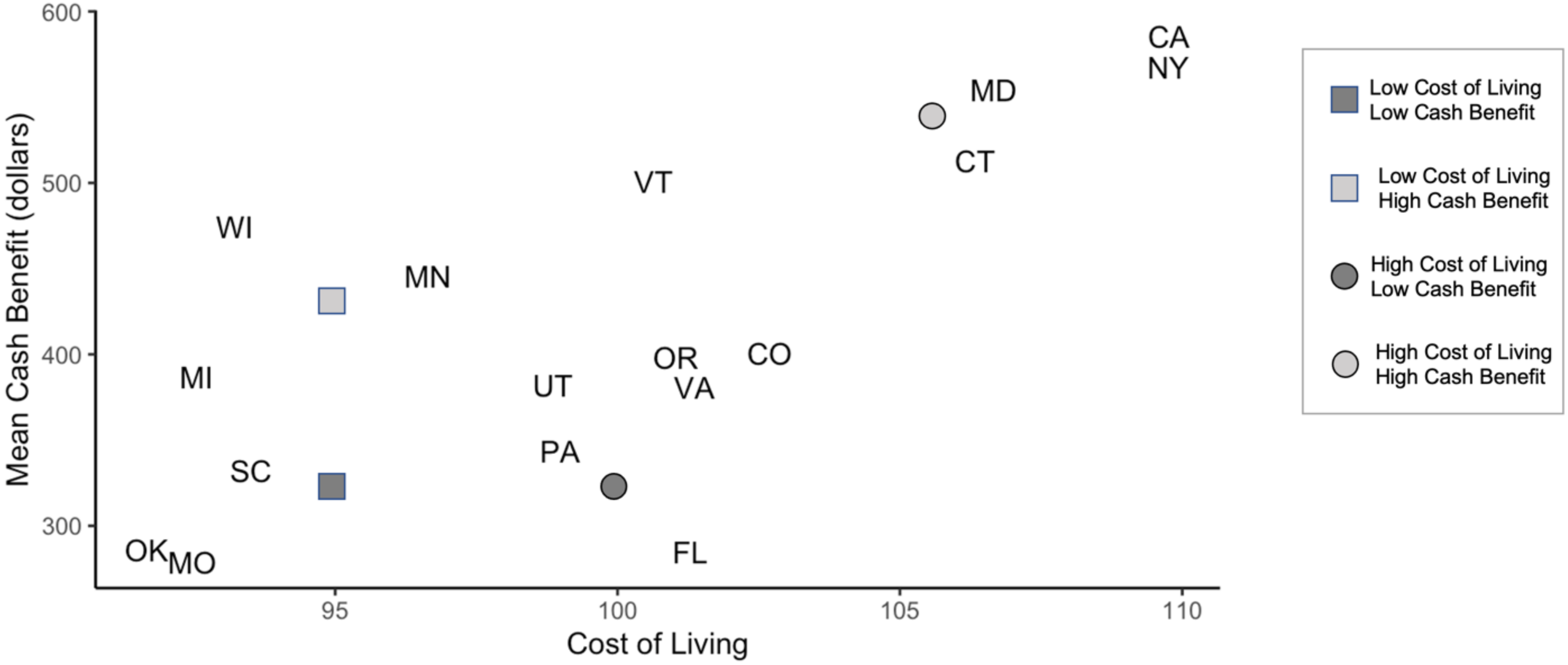

Figure S2. Distribution of Cost of Living Relative to Medicaid Expansion and Centering For Simple Slopes Analysis.

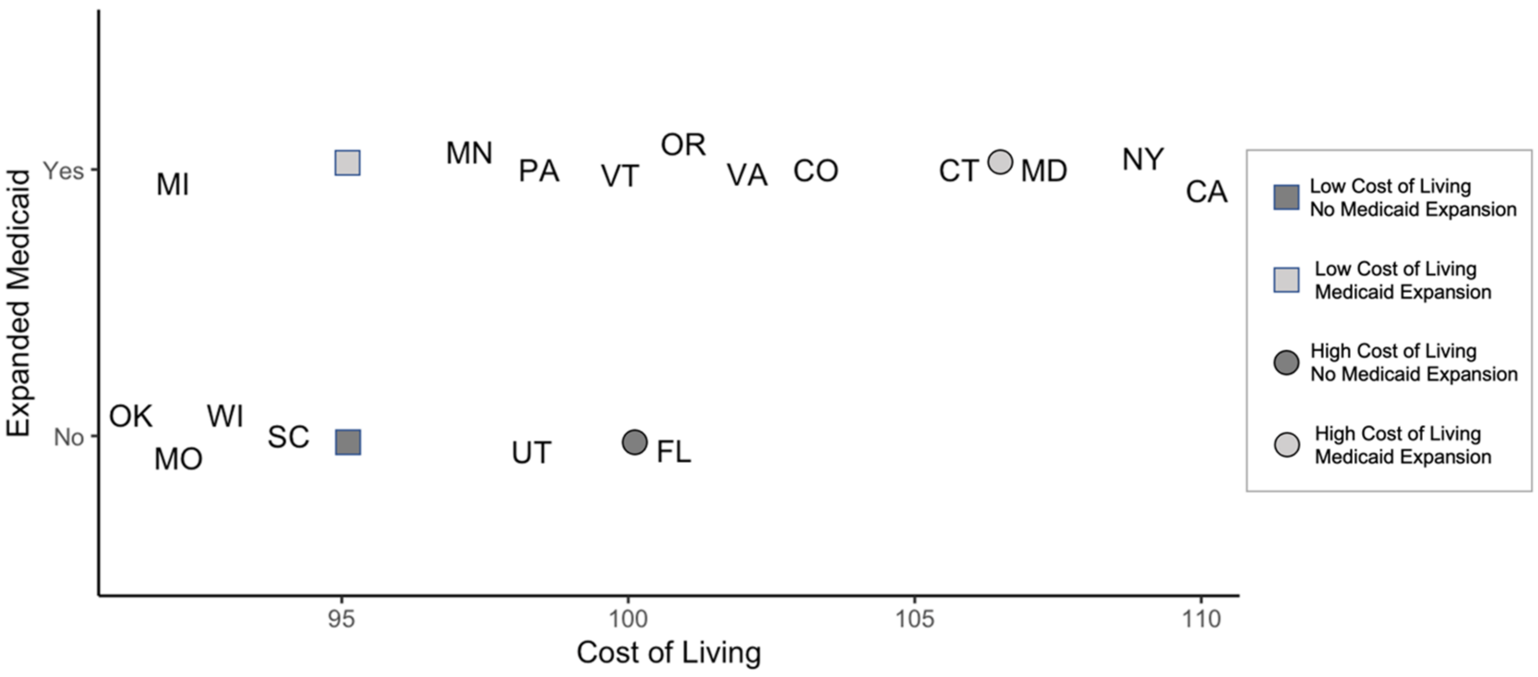

Supplemental References

1. U.S. Census Bureau. 2019 American Community Survey: 5-Year Estimates. https://data.census.gov/cedsci/table?q=ACSDT1Y2019.B19083&g=0100000US.04000.001_0400000US06,08,09,12,24,26,27,29,36,40,41,42,45,49,50,51,55&tid=ACSDT5Y2019.B19083&hidePreview=true (2020).
2. Gini, C. On the measure of concentration with special reference to income and statistics. *Colorado College Publication, General Series* **208**, 73–79 (1936).
3. U.S. Department of Labor. Changes in Basic Minimum Wages in Non-Farm Employment Under State Law: Selected Years 1968 to 2021 |. https://www.dol.gov/agencies/whd/state/minimum-wage/history (2022).
4. U.S. Bureau of Labor Statistics. Unemployment Rates for States. *Local Area Unemployment Statistics* https://www.bls.gov/lau/lastrk17.htm (2021).
5. Maruschak, L. M., Minton, T. D. & BJS Statisticians. *Correctional Populations in the United States, 2017-2018*. https://bjs.ojp.gov/library/publications/correctional-populations-united-states-2017-2018 (2020).
6. Rabuy, B. & Kopf, D. *Prisons of Poverty: Uncovering the pre-incarceration incomes of the imprisoned*. https://www.jstor.org/stable/resrep27310 (2015).
7. Friedman-Krauss, A. H. *et al. The State of Preschool 2017*. 347 (2018).
8. National Center for Education Statistics. *National Assessment of Educational Progress (NAEP), 2019 Reading Assessment*. (2020).
9. Federal Election Comission of the United States of America. *Official 2020 Presidential General Election Results*. https://www.fec.gov/resources/cms-content/documents/2020presgeresults.pdf.

10. 2020 presidential election results. https://www.cnn.com/election/2020/results/president.

11. Hess, C. *et al. The Status of Women in the States: 2015*. https://iwpr.org/iwpr-publications/report/the-status-of-women-in-the-states-2015/ (2015).

12. Harrington, J. R. & Gelfand, M. J. Tightness–looseness across the 50 united states. *PNAS* **111**, 7990–7995 (2014).
