# Supplementary material for: State-level macro-economic factors moderate the association of low income with brain structure and mental health in U.S. children
Source: Nat Commun. 2023 May 2;14:2085. doi: 10.1038/s41467-023-37778-1 (PMC10154403; doi:10.1038/s41467-023-37778-1)
Supplement: Supplementary file 3 — Reporting Summary [file 41467_2023_37778_MOESM3_ESM.pdf]

nature portfolio

Corresponding author(s): David G. Weissman

Last updated by author(s): 03/07/2023

Reporting Summary

Nature Portfolio wishes to improve the reproducibility of the work that we publish. This form provides structure for consistency and transparency in reporting. For further information on Nature Portfolio policies, see our Editorial Policies and the Editorial Policy Checklist.

Please do not complete any field with "not applicable" or n/a. Refer to the help text for what text to use if an item is not relevant to your study.
For final submission: please carefully check your responses for accuracy; you will not be able to make changes later.

Statistics

For all statistical analyses, confirm that the following items are present in the figure legend, table legend, main text, or Methods section.

n/a	Confirmed	
☐	☒	The exact sample size (*n*) for each experimental group/condition, given as a discrete number and unit of measurement
☐	☒	A statement on whether measurements were taken from distinct samples or whether the same sample was measured repeatedly
☐	☒	The statistical test(s) used AND whether they are one- or two-sided<br>*Only common tests should be described solely by name; describe more complex techniques in the Methods section.*
☐	☒	A description of all covariates tested
☐	☒	A description of any assumptions or corrections, such as tests of normality and adjustment for multiple comparisons
☐	☒	A full description of the statistical parameters including central tendency (e.g. means) or other basic estimates (e.g. regression coefficient) AND variation (e.g. standard deviation) or associated estimates of uncertainty (e.g. confidence intervals)
☐	☒	For null hypothesis testing, the test statistic (e.g. *F*, *t*, *r*) with confidence intervals, effect sizes, degrees of freedom and *P* value noted<br>*Give P values as exact values whenever suitable.*
☒	☐	For Bayesian analysis, information on the choice of priors and Markov chain Monte Carlo settings
☐	☒	For hierarchical and complex designs, identification of the appropriate level for tests and full reporting of outcomes
☐	☒	Estimates of effect sizes (e.g. Cohen's *d*, Pearson's *r*), indicating how they were calculated

Our web collection on statistics for biologists contains articles on many of the points above.

Software and code

Policy information about availability of computer code

Data collection	Data were obtained from the ABCD curated data release from the NIMH Data Archive using the NIMH Data Archive Download Manager.
Data analysis	Analyses were conducted using linear mixed-effects models with the nlme package in R version 4.0.0.

For manuscripts utilizing custom algorithms or software that are central to the research but not yet described in published literature, software must be made available to editors and reviewers. We strongly encourage code deposition in a community repository (e.g. GitHub). See the Nature Portfolio guidelines for submitting code & software for further information.

Data

Policy information about availability of data

All manuscripts must include a data availability statement. This statement should provide the following information, where applicable:

- Accession codes, unique identifiers, or web links for publicly available datasets
- A description of any restrictions on data availability
- For clinical datasets or third party data, please ensure that the statement adheres to our policy

Data is from the Adolescent Brain and Cognitive Development Study. Information on how to access ABCD data through the NIMH Data Archive (NDA) is available on the ABCD study data sharing webpage: https://abcdstudy.org/scientists_data_sharing.html. Deidentified data for the current analyses can be found at https://osf.io/t3ev7/.

Human research participants

Policy information about studies involving human research participants and Sex and Gender in Research.

Reporting on sex and gender	The analytic sample for the present study consists of 5,115 (48%) female and 5,518 (52%) male participants based on self-reported sex. Sex differences are commonly observed in hippocampal volume, internalizing problems, and externalizing problems, so sex was included as a covariate in all analyses. However, we have no reason to believe based on any theory or previous findings that the associations of family income with hippocampal volume, externalizing problems, or internalizing problems would vary as a function of sex. Moreover, as the proportion of males and females was roughly the same at every study site, there is no reason to believe that the extent to which the association between family income and each outcome varies as a function of state-level characteristics would differ between males and females. Therefore, all analyses were conducted in both males and females together.
Population characteristics	See below
Recruitment	The ABCD Study aimed to recruit a multi-stage probability sample of eligible youth by first selecting a stratified, probability sample of schools across the U.S. in order to capture demographic diversity. For each of the 21 ABCD sites, t a catchment area was defined as all schools within 50 miles of the research institution. Each school within the catchment area was coded according to geographical location, racial, ethnic and sex composition, and percentage of students receiving free or subsidized lunches. Based on this information, a stratified subset of schools was randomly selected from each catchment area. Procedures were used to ensure that systematic sampling biases in recruitment at the school level were minimized. The ABCD Study then recruited eligible children from each of the randomly selected schools within the catchment area. Initial recruitment often involved the delivery of hard and electronic copies of recruitment materials to caregivers. The ABCD Study sample was recruited in an epidemiologically informed manner to reduce selection bias. However, the degree to which this sample is fully representative of the U.S. population varies across the outcome measure examined.
Ethics oversight	Most ABCD research sites rely on a central Institutional Review Board (cIRB) at the University of California, San Diego for the ethical review and approval of the research protocol, with a few sites obtaining local IRB approval. The Coordinating Center is responsible for coordinating all interactions between relying sites and the cIRB. In addition, the Coordinating Center has established a Bioethics and Medical Oversight advisory group.

Note that full information on the approval of the study protocol must also be provided in the manuscript.

Field-specific reporting

Please select the one below that is the best fit for your research. If you are not sure, read the appropriate sections before making your selection.

☐ Life sciences ☒ Behavioural & social sciences ☐ Ecological, evolutionary & environmental sciences

For a reference copy of the document with all sections, see nature.com/documents/nr-reporting-summary-flat.pdf

Behavioural & social sciences study design

All studies must disclose on these points even when the disclosure is negative.

Study description	Quantitative correlational study.
Research sample	We drew data from the baseline assessment of 11,864 youth with data on parent-reported psychopathology and 11,533 youth with brain structure data (5,291 female, 47.9%, mean age 9.91 years, SD=.622). 52.1% White, 15.0% Black, 20.3% Hispanic, 2.1% Asian, and 10.5% other [e.g., biracial]
Sampling strategy	Twenty-one study sites were included from across the U.S. From these sites, a stratified probability sample of schools within the catchment areas for each site were selected, and eligible youth were recruited from each school. The ABCD study approximates a multi-stage probability sample, but is not nationally-representative. The imaging procedures were harmonized across sites. The ABCD study was designed to detect small effects the ABCD Study's to detect small effects. For example it can detect a Cohen's d of.30 with 90% power.
Data collection	Self- and parent-report measures were collected using ipads. MRI measures were collected using 3T MRI machines. The ABCD study was designed to address a multitude of potential research questions by making the data open to the public. Therefore, the researchers collecting the data were unaware of the specific questions or hypotheses of the current study.
Timing	Baseline data was collected between January 2017 and September 2018. 1-year follow-up data was collected between January and December 2018
Data exclusions	ABCD guidelines were followed with regard to exclusion of participants based on data quality, motion, or inattention during the MRI. This included exclusion of structural data that was rated as severe in any of the five categories of image artifact or reconstruction

	inaccuracy: motion, intensity inhomogeneity, white matter underestimation, pial overestimation, and magnetic susceptibility artifact. A total of 471 participants were excluded from further analyses based on these criteria.
Non-participation	11,864 parents of youth reported demographic information and on psychopathology. However, 1,236 of those parents declined to provide information about family income and were therefore excluded from these analyses. An additional 251 participants did not complete the MRI session and are therefore not included in analyses related to hippocampal volume.
Randomization	Participants were not allocated to experimental groups. The ABCD study is exclusively correlational.

Reporting for specific materials, systems and methods

We require information from authors about some types of materials, experimental systems and methods used in many studies. Here, indicate whether each material, system or method listed is relevant to your study. If you are not sure if a list item applies to your research, read the appropriate section before selecting a response.

Materials & experimental systems

n/a	Involved in the study
☒	☐ Antibodies
☒	☐ Eukaryotic cell lines
☒	☐ Palaeontology and archaeology
☒	☐ Animals and other organisms
☒	☐ Clinical data
☒	☐ Dual use research of concern

Methods

n/a	Involved in the study
☒	☐ ChIP-seq
☒	☐ Flow cytometry
☐	☒ MRI-based neuroimaging

Magnetic resonance imaging

Experimental design

Design type	na
Design specifications	na
Behavioral performance measures	na

Acquisition

Imaging type(s)	structural
Field strength	3T
Sequence & imaging parameters	The T1w acquisition (1 mm isotropic) is a 3D T1w inversion prepared RF-spoiled gradient echo scan using prospective motion correction, when available (currently only on Siemens and GE scanners) (Tisdall et al., 2012; White et al., 2010). The T2w acquisition (1 mm isotropic) is a 3D T2w variable flip angle fast spin echo scan, also using prospective motion correction when available.
Area of acquisition	whole brain

Diffusion MRI ☐ Used ☐ Not used

Preprocessing

Preprocessing software	Freesurfer 5.3
Normalization	na
Normalization template	na
Noise and artifact removal	na
Volume censoring	na

Statistical modeling & inference

Model type and settings	Hippocampal volume based on freesurfer autosegmentation was used as a dependent variable in analyses, controlling for estimated total intracranial volume.

Effect(s) tested	na

Specify type of analysis: [ ] Whole brain [x] ROI-based [ ] Both

Anatomical location(s)	Hippocampus
Statistic type for inference (See Eklund et al. 2016)	na
Correction	na

Models & analysis

n/a	Involved in the study
[x]	[ ] Functional and/or effective connectivity
[x]	[ ] Graph analysis
[x]	[ ] Multivariate modeling or predictive analysis

is checklist template is licensed under a Creative Commons Attribution 4.0 International License, which permits use, sharing, adaptation, distribution and reproduction in any medium or format, as long as you give ppropriate credit to the original author(s) and the source, provide a link to the Creative Commons license, and indicate if changes were made. The images or other third party material in this article are included in e article's Creative Commons license, unless indicated otherwise in a credit line to the material. If material is not included in the article's Creative Commons license and your intended use is not permitted by atutory regulation or exceeds the permitted use, you will need to obtain permission directly from the copyright holder. To view a copy of this license, visit http://creativecommons.org/licenses/by/4.0/
